# Specialists in ancient trees are more affected by climate than generalists

**DOI:** 10.1002/ece3.1799

**Published:** 2015-11-17

**Authors:** Leonie A. Gough, Anne Sverdrup‐Thygeson, Per Milberg, Hanne E. Pilskog, Nicklas Jansson, Mats Jonsell, Tone Birkemoe

**Affiliations:** ^1^ Department of Ecology and Natural Resource ManagementNorwegian University of Life SciencesP.O. Box 5003AasNO‐1432Norway; ^2^ Department of Life SciencesImperial College LondonSilwood Park CampusAscotBerkshireSL5 7PYUnited Kingdom; ^3^ IFM BiologyConservation Ecology GroupLinköping UniversityLinköpingSE‐581 83Sweden; ^4^ Department of EcologySwedish University of Agricultural SciencesBox 7044UppsalaSE‐750 07Sweden

**Keywords:** Beetles, climate gradient, coleoptera, precipitation, saproxylic, temperature

## Abstract

Ancient trees are considered one of the most important habitats for biodiversity in Europe and North America. They support exceptional numbers of specialized species, including a range of rare and endangered wood‐living insects. In this study, we use a dataset of 105 sites spanning a climatic gradient along the oak range of Norway and Sweden to investigate the importance of temperature and precipitation on beetle species richness in ancient, hollow oak trees. We expected that increased summer temperature would positively influence all wood‐living beetle species whereas precipitation would be less important with a negligible or negative impact. Surprisingly, only oak‐specialist beetles with a northern distribution increased in species richness with temperature. Few specialist beetles and no generalist beetles responded to the rise of 4°C in summer as covered by our climatic gradient. The negative effect of precipitation affected more specialist species than did temperature, whereas the generalists remained unaffected. In summary, we suggest that increased summer temperature is likely to benefit a few specialist beetles within this dead wood community, but a larger number of specialists are likely to decline due to increased precipitation. In addition, generalist species will remain unaffected. To minimize adverse impacts of climate change on this important community, long‐term management plans for ancient trees are important.

## Introduction

Ancient trees are keystone structures in natural, agricultural, and urban ecosystems around the globe (Vera 2000; Brawn 2006; Gibbons et al. 2008; Andersson et al. 2011; Lindenmayer et al. 2012; Buse et al. 2013; Bouget et al. 2014). In the northern temperate zone, ancient hollow oak trees (*Quercus* spp.) are considered one of the most important habitats for biodiversity, both in Europe (Vera 2000; Andersson et al. 2011; Bouget et al. 2014) and in North America (Brawn 2006). They support exceptional numbers of specialized biodiversity, including a range of rare and endangered species of wood‐living insects (Ranius and Jansson 2000; Buse et al. 2010; Sverdrup‐Thygeson et al. 2010). These specialist species have important ecological roles, particularly as decomposers (Ulyshen 2014) and as they tend to be rare in a community, the roles are often irreplaceable (Mouillot et al. 2013). They contribute to maintaining functional diversity, the importance of which for ecosystem services is increasingly acknowledged (Diaz and Cabido 2001; Díaz et al. 2007; Arnan et al. 2013). Specialists are known to be more negatively affected by habitat loss than are generalists (Slatyer et al. 2013), but their responses to climate change are little understood.

Climate change is an ever‐increasing threat to biodiversity (Bellard et al. 2012). Temperature has increased by 0.74°C and precipitation by 6–8% in the northern hemisphere during the last century alone (IPCC, 2007). Conservative projections of climate change estimate a further 2°C rise in temperature by 2100. But as recent greenhouse gas emission rates are the highest ever observed (World Meteorological Organization, 2014), we are more likely to experience a global temperature increase closer to the 4°C predicted under both constant and high emissions scenarios (IPCC, 2013). In tandem with these vast temperature changes, precipitation in the northern hemisphere is likely to increase over the coming decades (Kjellström et al. 2011).

In response to the recent temperature changes, bark beetles have expanded to higher elevations and latitudes beyond their historic limits (Logan et al. 2003; Parmesan 2006). Butterflies, representing the best‐known insect group, appear several weeks earlier than three decades ago on various continents (Roy and Sparks 2000; Forister and Shapiro 2003; Diamond et al. 2011). Multibrooded species have longer flight periods (Roy and Sparks 2000), and some bark beetles, butterflies, and moths have shifted from one to several generations in warmer summers (Jönsson et al. 2009; Altermatt 2010). Compared to temperature, few studies have focused on the possible effects of increased precipitation on insect communities. Existing studies have found that high precipitation may limit insect flight ability (Bale et al. 2002; Klueken et al. 2009; Jaramillo et al. 2011; Sturtevant et al. 2013), but it is not possible to anticipate effects at a wider community level.

Despite their importance for ecosystem functioning and high proportion of threatened species, the effect of climate on the dead wood beetle community is little studied. There is some indication that climate will affect species richness. Temperature has been found to be a predictor of dead wood‐living beetle species richness along geographical and altitudinal gradients in Europe (Gossner et al. 2013; Müller et al. 2014). Precipitation has been found to have either no effect on species richness (Müller et al. 2014) or a mixed response, depending on functional group (Gossner et al. 2013). However, it is not known how these effects vary between host tree species, and whether specialists and generalists respond in the same way. Species may respond quite differently in oak as opposed to other host trees such as beech, and in ancient, natural forest to managed productive woodland. Specialists and generalists are known to respond differently to habitat change and so may respond differently to climate change. Specialists might experience larger challenges from temperature change alone or in combination with habitat degeneration than generalists (Stefanescu et al. 2011; Filz et al. 2013; Ball‐Damerow et al. 2014). Specialists might be more sensitive than generalists to precipitation (Leckey et al. 2014).

Geographical range is also important. The current geographical range of a species indicates the climate regime to which it is adapted. Also, the size of a species geographical range often correlates with niche‐breadth; the larger the range, the wider the niche‐breadth (Slatyer et al. 2013). Taking geographical range into account when analyzing species responses to climate may reveal important patterns that are otherwise overlooked. Thus, to predict future changes in species diversity and subsequently ecosystem services, specialist and generalist species ought to be treated separately and geographical range taken into account. With the prospect of rapid and sustained change in both temperature and precipitation, it is important to understand how specialist and generalist wood‐living insects may respond to inform conservation and begin to understand the implications for the services they provide.

In this study, we use a huge dataset of 105 sites – spanning a climatic gradient that mimics the temperature increase of 4°C and precipitation changes likely to occur to 2100 along the oak range of Norway and Sweden – to investigate the likely consequences of future climate change for the species richness of wood‐living beetles in ancient, hollow oak trees. We examine specialists and generalists, and those with differing geographical ranges separately. We expect that (1) increased summer temperature will positively influence all wood‐living beetle species and in particular, those with a southern distribution and (2) precipitation may be less important than temperature, with a negligible or negative impact.

## Materials and Methods

### Data collection

**Figure 1:**
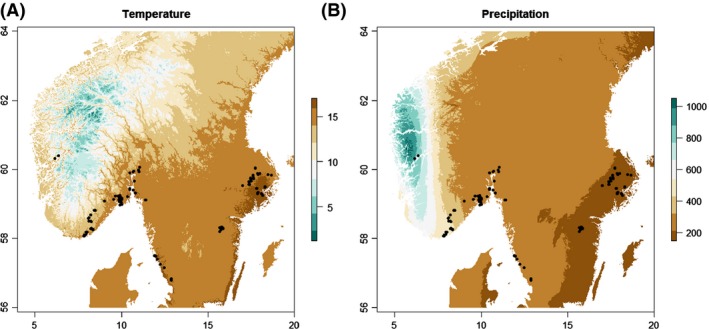
Study sites location with (A) mean temperature during warmest quarter of the year and (B) mean precipitation during warmest quarter of the year in millimeter. Climate data were downloaded from BIOCLIM (variables BIO10 and BIO18) (Hijmans et al. 2005). Axes are longitude and latitude.

The study contains data from 308 hollow oak trees at 105 sites, spread along a 700‐km climatic gradient mainly going from west to east across southern Norway and Sweden (Fig. 1). The gradient covers a range of 4°C (12.5–16.9°C) difference in temperature during the warmest quarter of the year. As for precipitation, there is a 620‐mm difference between the highest (798 mm) and lowest (178 mm) values in our study sites (Fig. 1). Excluding the two westernmost sites, the range spans 303 mm.

The sites fall within the hemiboreal zone (Ahti et al. 1968) which, although dominated by boreal tree species, is also characterized by a considerable element of southern deciduous tree species, for example oak *Quercus robur* and *Quercus petraea,* lime *Tilia cordata*, maple *Acer platanoides*, ash *Fraxinus excelsior*, elm *Ulmus glabra*, and hazel *Corylus avellana*, on richer soils and on sites with a warmer microclimate.

We surveyed all sites during 1 year in the period 1999–2009. We sampled one to four hollow oaks (defined as having a minimum of 60‐cm circumference at breast height with a visible cavity in the trunk) at each site (Table S1). Oaks were randomly chosen within a site. Where more than one tree was sampled at a site, trees were between 6 m and 250 m apart. Sites were at least 1500 m apart. Tree age was not known precisely, but the age of a subset of the Swedish oaks was estimated to be 150–500 years old. Each tree was sampled for beetles using one flight interception (window) trap with a transparent plastic window above a container of preservative solution and detergent to break surface tension. Trap windows were either 20 × 40 cm or 30 × 50 cm and placed as close to the cavity opening as possible (within 1 m). Height above the ground varied from 1.5 m to 7 m (average 2–3 m) dependent on the height of the cavity opening. They were active during the summer beetle flight season from May to September, and emptied once per month. The sites were originally surveyed for slightly different purposes, but all for wood‐living beetles and the sampling methods were consistent across sites and years. The tree selection and sampling process for all sites is described in detail in Jansson et al. (2009), Sverdrup‐Thygeson et al. (2010), Pilskog et al. (2014), Jonsell and Eriksson (2002) and Jonsell (2008).

The original surveys had slightly different taxonomic ambition (i.e. ignoring some groups). However, for all sites, beetle species known to be associated with oak were identified, and based on this criterion, we made a common species list of 152 species that was comparable across sites (Table S2). We classified the species into specialists and generalists according to their association with oak trees based on an open database on host tree association from Fennoscandia (The Saproxylic Database, 2014). Specialists were defined as species occurring primarily on dead wood from oak and occasionally on other broad‐leaved temperate species and generalists as species occurring on dead wood from a range of trees (deciduous and coniferous), including oak (see Table S2 and The Saproxylic Database for classification details). Species not associated with oak at all were excluded from the analysis. In addition, the species were categorized according to their geographical distribution; Southern species, occurring primarily in the south of Europe (south of ~47 degrees N); Northern species, occurring primarily in the north of Europe (north of ~50 degrees N); and Widespread species which do not fall into either group and are widespread. The Widespread group also includes a few species with a central European distribution. The distribution was based on the European Nature Information System (eunis.eea.europa.eu), Global Biodiversity Information Facility (www.gbif.org), Fauna Europaea (www.faunaeur.org), Encyclopedia of Life (www.eol.org), and various other resources depending on species. This resulted in six species groups: (1) Northern Specialists, (2) Southern Specialists, (3) Widespread Specialists, (4) Northern Generalists, (5) Southern Generalists, and (6) Widespread Generalists (Table S2). We summed the species counts per group and tree.

We chose to use an open database (Dahlberg and Stokland 2004; The Saproxylic Database, 2014) on host tree association to classify the species' host tree dependence. However, we found that the classification did not fully correspond with our own experience of beetle specialization. To test the results' sensitivity to the classification, we revised the original classification based on our field sampling experiences of oak and other host trees (Table S2) and compared the importance of our explanatory variables to the response variables based on univariate statistics with the two classifications. The new classification gave similar but stronger results for the specialist species and similar but weaker results for the generalists. Few species were categorized as Widespread in the new classification, so the comparison was inconclusive for these species.

Two climate variables and two environmental variables were included in all analyses. Climate data were derived from BIOCLIM (Hijmans et al. 2005). We chose the variables BIO10 Mean Temperature during the Warmest Quarter of the Year and BIO18 Mean Precipitation during the Warmest Quarter of the Year as the most relevant climate measures for beetle development and swarming (Fig. 1). We extracted the data for each tree using the extract function in the “raster” package in R (Hijmans 2014) and we used the “RColorBrewer” package (Neuwirth 2014) to visualize the changes in temperature and precipitation across the gradient (Fig. 1). To account for additional variation in species richness, we also included Circumference of the sampled hollow oak (cm at breast height, measured with a tape) and Openness immediately around the oak (0 = open, 1 = partly closed canopy, 2 = closed canopy). These are ecologically meaningful variables known to affect wood‐living beetle species richness (Ranius and Jansson 2000; Sverdrup‐Thygeson et al. 2010; Gough et al. 2014). Openness reflects the amount of surrounding vegetative growth and the amount of sunlight that reaches the trunk (and hence cavity) and crown of the oak. The openness around the oak tree has been found to be the most important factor for wood‐living beetle species richness in previous work (Gough et al. 2014), and it provides an insight into the microclimate of the tree within the broader climate gradient.

### Statistical analysis

All analyses were carried out in R version 3.1.0 (R Core Team, 2014). We used species richness per tree within each category of species as a response variable. To evaluate whether temperature and precipitation influence species richness, we applied generalized linear mixed‐effects models (GLMMs) using function glmer from package “lme4” (Bates et al. 2014) with a Poisson error distribution, log‐link function, and Nelder–Mead optimizer from package “nloptr” (Nelder and Mead 1965; Johnson 2014). Continuous variables (Circumference, Temperature, and Precipitation) were checked for collinearity through calculation of correlation coefficients and inspection of variance inflation factors (VIF), then centered and scaled. We used Site as a random effect to account for large variances between species richness of different sites, and to allow generalization of the results beyond the sampled trees. Country (Norway/Sweden) was used as a random effect to account for large variances between the species richness of the different countries. To validate model fit, we checked overdispersion of the residuals for each species group (it was between 0.9 and 1 for all groups) and visually assessed the distribution of the residuals using QQ plots and plots of residual against fitted values. An additional analysis using backwards stepwise selection by use of the drop1 function, based on Akaike's Information Criterion (AIC), was carried out to select the most parsimonious model for each species group (Table S3).

## Results

The dataset we used consisted of 3036 occurrences of 152 beetle species. For generalists, there were an even number of Northern and Southern species (51 Northern, 52 Southern). It was highly uneven for specialists, with four times as many Southern Specialists (24 species) as Northern Specialists (six species) in total, although the mean, minimum, and maximum number of Northern Specialists and Southern Specialists per tree was identical. There were few Widespread species, with a mode of zero counts per tree and a mean of less than one species per tree (Table 1).

**Table 1:** Number of species per group

Species group	Total number of species	Mean richness per tree (min, max)
Northern specialists	6	1.1 (0, 5)
Southern specialists	24	1.1 (0, 5)
Widespread specialists	7	0.7 (0, 4)
Northern generalists	51	3.9 (0, 14)
Southern generalists	52	2.5 (0, 13)
Widespread generalists	12	0.6 (0, 3)

### Climate

**Figure 2:**
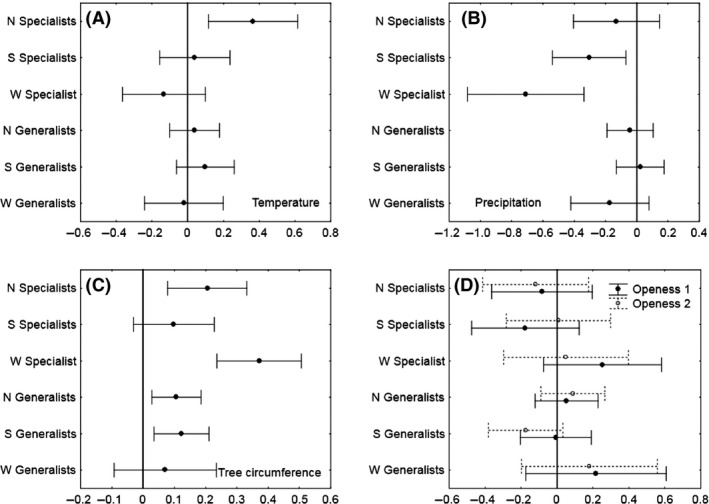
Partial regression coefficients (with 95% CI) from six regression models, one for each species group (Generalist and Specialists divided into Northern (N), Southern (S), and Widespread (W) species). In the models, the richness was predicted by summer temperature (A), summer precipitation (B), hollow oak circumference (C), the openness of the immediate surroundings of a tree (D); 1 = partly closed canopy, 2 = closed canopy. 0 = open canopy, used as the baseline in model estimates.

The species richness of all three specialist groups (Northern, Southern, and Widespread) was significantly affected by either Temperature (positive) or Precipitation (negative). In contrast, no generalist group was affected by a climate variable (Fig. 2).

The effect of Temperature was significant for only one group, the Northern Specialists, for which species richness increased with Temperature (Fig. 2A). Precipitation overall had a stronger impact than Temperature, as it negatively affected Southern as well as Widespread Specialists (Fig. 2B). No group was significantly affected by both Temperature and Precipitation. This pattern, and Precipitation having an effect of larger magnitude than Temperature, was confirmed and even strengthened, in the most parsimonious models; the Northern Specialists were the only model that contained temperature (significant positive effect), while three models – Southern and Widespread Specialists and Widespread Generalists – contained Precipitation (negative effect) (Table S3). Precipitation was, however, not significant (*p* = 0.86) in the model with Widespread Generalists.

As the results for specialists were found to be strengthened under an alternative classification (see Methods), we consider the results presented here to be a conservative estimate of the effect of temperature and precipitation on Specialists.

### Environmental variables

Every group except Widespread Generalists was significantly affected by the size of the tree, with species richness increasing as Circumferences increases (Fig. 2C, Table S3). Openness is known to be very important for hollow oak beetle species and was a significant variable when tested alone (results not shown), but did not significantly affect any group in the full models (Fig. 2D). Openness was removed from all models early in the selection process during stepwise selection and was not included in any of the most parsimonious models (Table S3).

## Discussion

Our study of beetle species richness in ancient trees along a climate gradient provides a starting point to understand and predict wood‐living beetles' responses to climate change. Our results indicate that increased summer temperature and summer rain could substantially affect wood‐living beetle species richness and that the effect is likely to be more profound for oak specialists than generalists.

### Effect of increased mean summer temperature

Our specialist beetle species are primarily found on *Q. robur* and *Q. petraea*, which need warm, sunny conditions to successfully grow to a great age (Vera 2000). The beetles are therefore likely to be adapted to stable and warm conditions, and we expected their species richness to increase with temperature independent of their present geographical distribution. Such patterns have been demonstrated for wood‐living beetles in other European studies. In a study of beech forest, Müller et al. (2014) found an increase in species richness of wood‐living beetles along temperature gradients in Europe. A recent report also concluded that species richness of wood‐living beetles has increased during the last 20 years, probably as a result of global warming (Köhler 2014). Contrary to our expectation, our study did not confirm this pattern for the majority of the species; only specialist species with a Northern distribution responded positively to temperature in our gradient.

It is difficult to untangle the underlying processes determining the richness of hollow oak species along temperature gradients. Some explanation might be related to the fact that specialists, with their narrower habitat demands compared to generalists, may be particularly sensitive to additional constrains such as biotic interactions which may interfere with climate predictions (Preston et al. 2008). Hollow tree specialists were also found to be less affected by global warming than the total species richness reported by Köhler (2014). This could be due to a potential buffering effect of living within a tree cavity, where, depending on the size of the cavity and amount of wood‐mold within it, temperatures tend to be more stable than outside or in small pieces of dead wood (Ranius and Nilsson 1997). The responses of the specialist hollow oak beetles could therefore be modified by the immediate microclimate of the hollow. In order to understand the contrasting responses, more detailed studies are clearly needed.

The temperature range of our study, a difference of 4°C, corresponds to the predicted temperature rise in the study region of 4.6 degrees by 2100 (Hanssen‐Bauer et al. 2009). Thus, our findings along the geographical gradient may potentially describe the changes to be expected toward the end of this century – acting as a warning of possible changes in abundance patterns in the important community of beetles in ancient trees in Northern Europe.

### Effect of increased mean summer precipitation

We expected that precipitation would have a negligible or possibly negative effect on species richness. This was confirmed, with no effect for four and negative effect for two (both specialists) of the six species groups in the hollow oaks.

It is likely that precipitation reduces flight time and dispersal distance, as insects may fly less during wet periods (Klueken et al. 2009; Jaramillo et al. 2011). This could shorten or interrupt the overall flight period and disrupt dispersal and colonization of new trees. The Southern and Widespread specialists responded most strongly to precipitation. Possibly, they are less likely to fly in rain than the other beetles, as they generally live in drier and warmer climates. However, the importance of indirect effects should not be overlooked. Changes in wood‐rot fungi might be of particular importance, as many beetle species in dead wood interact with the fungal community (Crowson 1981; Müller et al. 2002), and fungi generally are sensitive to changes in moisture. Gossner et al. (2013) found an increase in the proportion of fungivorous beetle species in dead wood with increasing precipitation in Germany, whereas fresh wood feeders decreased. Thus, it is possible that our observed decrease in species richness among two specialist groups could be at least partly explained by changes in fungi‐interacting beetles along the gradient. However, two genera of known fungi‐interacting beetles in our study, *Cryptophagus* and *Mycetophagus,* were classified as generalists in groups that overall did not respond to precipitation, in contrast to Gossner et al.'s result. It is likely that the ecology and relationships between hollow oak species are highly complex and there are interactions taking place that are not yet understood. This highlights an advantage of studying wood‐living beetles at the overall community level. Indications that two specialist groups may be negatively affected by precipitation can guide future work to elicit the mechanisms behind the response, while still informing wider wood‐living beetle ecology and conservation, before individual species' responses can be studied in detail. In contrast to Gossner et al. (2013) and in line with most species in the present study, Müller et al. (2014) found no effect of precipitation on wood‐living beetle richness across European beech forests.

In the study area, precipitation is expected to increase by 5–30% by 2100 and most additional precipitation is predicted to occur in the already wet areas (Hanssen‐Bauer et al. 2009). As the proportional changes will be smaller in the dry as to compared to the wettest areas, the precipitation gradient is likely to be extended within the same geographical region and the specialist species richness may consequently decline even more toward the wetter end of the gradient.

Extreme climate events are likely to happen more frequently as the climate changes, with a potential for both increased drought and extreme precipitation events (IPCC, 2007). Whilst we did not examine the effect of extreme events, the results may provide some indication of their consequences. Drought with a lack of summer precipitation would not be likely to directly influence species richness of hollow oak beetle species, although it could be perceived as beneficial for the two specialist groups that were negatively affected by higher precipitation. It would likely affect the cavity microclimate and fungal community as discussed above. A greater impact could occur through the effects of a sustained drought on the host trees. Ancient trees are already in decline (Paillet et al. 2010; Lindenmayer et al. 2012), and drought can be a major stress contributing to oak tree aging and death (Vera 2000; Allen et al. 2010). Extreme precipitation events in the summer with intense periods of summer rain could affect beetle flight and enhance the negative effect observed in two groups. However, the contrasting response between groups and to climate and precipitation indicates that responses to extreme climate events are likely to be difficult to predict.

Trap catches are always a combination of population density and insect activity. Generally, insects are more flight active in warmer and dry weather as compared to cold and wet weather. Thus, one might argue that the coldest and wettest sites are being undersampled compared with warmer sites; it would take more trapping effort to catch beetles flying infrequently. However, if the results had been an artifact of sampling, we would expect a similar decline of species richness with high precipitation and low temperature in all species groups, but this was not observed. Müller et al. (2014) also found a consistent effect of temperature and precipitation on species richness comparing trapping data to data based on hatching from log units along a climatic gradient. Thus, it is unlikely that our findings are a result of a sampling bias.

### Environmental variables

Circumference and openness were included in the analyses as they are known to influence hollow oak beetle richness (Ranius and Jansson 2000). Circumference was confirmed as important with a significant positive effect for all species groups, likely related to the greater range and availability of habitat in the largest trees, and was retained in the most parsimonious model for all species groups except one (Widespread Generalists). However, the positive effect of circumference does not negate the effect of precipitation. The magnitude of the negative effect of precipitation was greater than the positive effect of circumference (Fig 2B,C). This means that even oak specialists in the “best trees” and “best quality habitat” are likely to be adversely affected by increased summer rain.

Openness, although significant alone and known to be important for beetle species composition (Lindhe et al. 2005; Vodka et al. 2009; Gough et al. 2014), did not affect species richness in the full models. Openness is a measure of how open or shaded the hollow oak tree is, and is a proxy for microclimate of the oak hollow. Thus, it is possible that the changes in species richness due to openness around the oak are eclipsed by a more profound gradient in climate. The result for openness in these models emphasizes the complex nature of climate change effects, and the synergy between climate and habitat change.

We did not include any measure of dead wood quantity or forest history in our analysis as this was only available for subsets of the entire dataset and measures used at different sites were not comparable. As the extent and abundance of ancient oaks in Sweden in recent times have been higher than in Norway (Eliasson and Nilsson 2002; Direktoratet for naturforvaltning, 2012), country was used as a random variable in all models as a proxy for forest history to account for any effect this may have had. This difference between Norway and Sweden in ancient oak abundance is driven by human activity and oak protection measures rather than climate (Direktoratet for Naturforvaltning, 2012), and so we find it unlikely that dead wood amount and forest history correlate strongly with the temperature and precipitation gradients in this particular study. However, we acknowledge that some variation within the dataset is likely to be explained by these variables and it would be beneficial to include them in future work.

### Potential impacts of future climate change

Our results indicate that with predicted climate change, only the specialists in hollow oaks with a Northern distribution are likely to benefit overall, as they were positively affected by temperature and did not respond to precipitation. Most specialists are likely to decline as they are negatively affected by precipitation and not affected by temperature, whereas generalists are likely to remain unchanged. Still, species expanding at the margins of their range may evolve greater dispersal abilities than those in the core (Hill et al. 2011; Lindstrom et al. 2013). Such an expansion cannot be ruled out and may potentially counteract the negative effect of precipitation on the Southern Specialist and increase species richness of Southern Generalists.

In general, hollow oak specialist beetles will be far more affected than generalists by climate change, and we may face an overall decrease in species richness in this community in Northern Europe. Although the specialists make up a relatively small species group compared to the generalists, they might be proportionally more important. They are often locally rare species, and as such often have functional traits with low redundancy, meaning that other species cannot easily replace their role if lost from the ecosystem (Mouillot et al. 2013).

The results discussed above assume that hollow oak habitats remain stable, which is questionable. Large, hollow oaks are considered to be in decline in Norway and Sweden due to direct removal, mechanical damage, and competition from dense surrounding growth (Naturvårdsverket, 2004, Sverdrup‐Thygeson et al. 2014). Action Plans are now in place in both countries aimed at halting the decline (Direktoratet for Naturforvaltning 2012, Naturvårdsverket, 2012). It is of course an open issue whether these attempts will impact the future abundance of large, hollow oaks and hence whether the possible adverse impacts of climate will be exacerbated. In any case, it will take several decades for the results of the Action Plans to become apparent due to the long lifespan of oak trees, and immediate (next 20+ years) climate change is not likely to be mitigated by a habitat increase.

Oak ecosystems in general are suffering a drastic decline worldwide (Vera 2000; Bjorkman and Vellend 2010; Paillet et al. 2010; Horak et al. 2014), and large, hollow trees are often disproportionately affected (Lindenmayer et al. 2014). Given the overall inability of hollow oak species to respond positively to temperature and their possible negative response to precipitation, a decline in the number of rare and threatened species is likely.

To conclude, the responses of wood‐living beetles to climate change are likely to be complex and to vary with geographical distribution and specialization. For hollow oak species, increased summer temperature is likely to benefit a few oak specialists with a northern distribution, but the majority of specialists is likely to decline due to increased precipitation. Oak generalist beetles are expected to remain unaffected. To minimize adverse impacts and allow for positive responses to climate change, it is important to halt the decline in ancient trees and consider restoration efforts that can maximize the quantity and quality of available habitat.

## Conflict of Interest

None declared.

## Supporting information


**Table S1.** Location of all 308 oaks from 105 sites sampled for beetles in the present study.

**Table S2.** Categorization of dead wood inhabiting beetles species according to oak association and primary geographical distribution.

**Table S3.** Results of backwards stepwise selection of six models all including four predictor variables: Temperature, Precipitation, Oak Circumference and Openness around trees.
