# Supplementary material for: Specialists in ancient trees are more affected by climate than generalists
Source: Ecol Evol. 2015 Nov 17;5(23):5632–41. doi: 10.1002/ece3.1799 (PMC4813105; doi:10.1002/ece3.1799)
Supplement: Supplementary file 1 — Table S1. Location of all 308 oaks from 105 sites sampled for beetles in the present study. [file ECE3-5-5632-s001.doc]

Table S1. Location of all 308 oaks from 105 sites sampled for beetles in the present study.

| Country | Site | OakID | UTM33_X | UTM33_Y | Responsible Author |
| --- | --- | --- | --- | --- | --- |
| Norway | Vollebekk/Korsegården | AA1 | 260867 | 6621558 | Anne Sverdrup-Thygeson |
| Norway | Årosveten | AR1 | 77806 | 6458628 | Anne Sverdrup-Thygeson |
| Norway | Årosveten | AR2 | 77683 | 6458842 | Anne Sverdrup-Thygeson |
| Norway | Årosveten | AR3 | 77638 | 6458847 | Anne Sverdrup-Thygeson |
| Norway | Årosveten | AR4 | 77608 | 6458967 | Anne Sverdrup-Thygeson |
| Norway | Årosveten | AR5 | 77684 | 6459096 | Anne Sverdrup-Thygeson |
| Norway | Askedalsåsane | AS1 | 207211 | 6558501 | Anne Sverdrup-Thygeson |
| Norway | Bøhler, Gjelleråsen | BG1 | 274885 | 6656350 | Anne Sverdrup-Thygeson |
| Norway | Berge landskapsvernområde | BL1 | 12946 | 6718980 | Anne Sverdrup-Thygeson |
| Norway | Berge landskapsvernområde | BL2 | 12936 | 6719005 | Anne Sverdrup-Thygeson |
| Norway | Berge landskapsvernområde | BL3 | 12901 | 6718952 | Anne Sverdrup-Thygeson |
| Norway | Berge landskapsvernområde | BL4 | 12858 | 6718864 | Anne Sverdrup-Thygeson |
| Norway | Berge landskapsvernområde | BL5 | 12819 | 6718853 | Anne Sverdrup-Thygeson |
| Norway | Bog (ved Carlberg) | BO1 | 253816 | 6592437 | Anne Sverdrup-Thygeson |
| Norway | Brenndalsskarven | BR1 | 206851 | 6578045 | Anne Sverdrup-Thygeson |
| Norway | Brenndalsskarven | BR2 | 206846 | 6578019 | Anne Sverdrup-Thygeson |
| Norway | Brenndalsskarven | BR3 | 206876 | 6578016 | Anne Sverdrup-Thygeson |
| Norway | Brenndalsskarven | BR4 | 206958 | 6578016 | Anne Sverdrup-Thygeson |
| Norway | Brenndalsskarven | BR5 | 206828 | 6578102 | Anne Sverdrup-Thygeson |
| Norway | Budalsåsen | BU1 | 215785 | 6566196 | Anne Sverdrup-Thygeson |
| Norway | Budalsåsen | BU2 | 215804 | 6566182 | Anne Sverdrup-Thygeson |
| Norway | Budalsåsen | BU3 | 215755 | 6566302 | Anne Sverdrup-Thygeson |
| Norway | Budalsåsen | BU4 | 215638 | 6566262 | Anne Sverdrup-Thygeson |
| Norway | Budalsåsen | BU5 | 215643 | 6566317 | Anne Sverdrup-Thygeson |
| Norway | Fjellstad (Asbjørnseneika) | FJ1 | 280218 | 6664849 | Anne Sverdrup-Thygeson |
| Norway | Gangseid | GA1 | 123223 | 6537857 | Anne Sverdrup-Thygeson |
| Norway | Gangseid | GA2 | 123196 | 6537853 | Anne Sverdrup-Thygeson |
| Norway | Gangseid | GA3 | 123190 | 6537816 | Anne Sverdrup-Thygeson |
| Norway | Gangseid | GA4 | 123214 | 6537943 | Anne Sverdrup-Thygeson |
| Norway | Gangseid | GA5 | 123221 | 6537942 | Anne Sverdrup-Thygeson |
| Norway | Gjønnesvannet | GJ1 | 214657 | 6569733 | Anne Sverdrup-Thygeson |
| Norway | Karljohansvern | KJ1 | 244248 | 6595798 | Anne Sverdrup-Thygeson |
| Norway | Karljohansvern | KJ2 | 244272 | 6595842 | Anne Sverdrup-Thygeson |
| Norway | Karljohansvern | KJ3 | 244243 | 6595743 | Anne Sverdrup-Thygeson |
| Norway | Karljohansvern | KJ4 | 244270 | 6595730 | Anne Sverdrup-Thygeson |
| Norway | Karljohansvern | KJ5 | 244185 | 6595730 | Anne Sverdrup-Thygeson |
| Norway | Knardal | KN1 | 293303 | 6558409 | Anne Sverdrup-Thygeson |
| Norway | Knardal | KN2 | 293281 | 6558471 | Anne Sverdrup-Thygeson |
| Norway | Knardal | KN3 | 293309 | 6558516 | Anne Sverdrup-Thygeson |
| Norway | Knardal | KN4 | 293343 | 6558500 | Anne Sverdrup-Thygeson |
| Norway | Knardal | KN6 | 293678 | 6558433 | Anne Sverdrup-Thygeson |
| Norway | Eikvang (Kjoseeika) | KO1 | 208960 | 6563444 | Anne Sverdrup-Thygeson |
| Norway | Kvelderønningen | KR1 | 210364 | 6573419 | Anne Sverdrup-Thygeson |
| Norway | Kvelderønningen | KR2 | 210167 | 6573730 | Anne Sverdrup-Thygeson |
| Norway | Kurland | KU1 | 277665 | 6650544 | Anne Sverdrup-Thygeson |
| Norway | Store Limtjønn | LI1 | 210047 | 6572601 | Anne Sverdrup-Thygeson |
| Norway | Melsomvik | ME1 | 234339 | 6574060 | Anne Sverdrup-Thygeson |
| Norway | Melsomvik | ME2 | 234286 | 6574111 | Anne Sverdrup-Thygeson |
| Norway | Melsomvik | ME3 | 234266 | 6573868 | Anne Sverdrup-Thygeson |
| Norway | Melsomvik | ME4 | 234473 | 6573642 | Anne Sverdrup-Thygeson |
| Norway | Melsomvik | ME5 | 234466 | 6573616 | Anne Sverdrup-Thygeson |
| Norway | Montebello | MO1 | 257996 | 6651634 | Anne Sverdrup-Thygeson |
| Norway | Montebello | MO2 | 257972 | 6651646 | Anne Sverdrup-Thygeson |
| Norway | Montebello | MO3 | 257953 | 6651587 | Anne Sverdrup-Thygeson |
| Norway | Montebello | MO4 | 257953 | 6651625 | Anne Sverdrup-Thygeson |
| Norway | Montebello | MO5 | 257962 | 6651606 | Anne Sverdrup-Thygeson |
| Norway | Østøya | OO1 | 243320 | 6598057 | Anne Sverdrup-Thygeson |
| Norway | Østøya | OO2 | 243310 | 6598038 | Anne Sverdrup-Thygeson |
| Norway | Østøya | OO3 | 243223 | 6598287 | Anne Sverdrup-Thygeson |
| Norway | Østøya | OO4 | 243283 | 6598373 | Anne Sverdrup-Thygeson |
| Norway | Østøya | OO5 | 243212 | 6598402 | Anne Sverdrup-Thygeson |
| Norway | Pauler | PA1 | 210086 | 6557777 | Anne Sverdrup-Thygeson |
| Norway | Ris (Risbakken 22) | RB1 | 259883 | 6653023 | Anne Sverdrup-Thygeson |
| Norway | Søndre Odbergsetra (Rimstad) | RI1 | 209614 | 6578345 | Anne Sverdrup-Thygeson |
| Norway | Sandvikskollane | SA1 | 207040 | 6569131 | Anne Sverdrup-Thygeson |
| Norway | Sandvikskollane | SA2 | 206601 | 6569019 | Anne Sverdrup-Thygeson |
| Norway | Simonstona | SI1 | 121002 | 6537596 | Anne Sverdrup-Thygeson |
| Norway | Simonstona | SI2 | 121018 | 6537765 | Anne Sverdrup-Thygeson |
| Norway | Simonstona | SI3 | 121079 | 6537808 | Anne Sverdrup-Thygeson |
| Norway | Simonstona | SI4 | 121112 | 6537824 | Anne Sverdrup-Thygeson |
| Norway | Simonstona | SI5 | 121080 | 6537755 | Anne Sverdrup-Thygeson |
| Norway | Skeianes | SK1 | 24179 | 6727712 | Anne Sverdrup-Thygeson |
| Norway | Skeianes | SK2 | 24185 | 6727746 | Anne Sverdrup-Thygeson |
| Norway | Skeianes | SK3 | 24202 | 6727756 | Anne Sverdrup-Thygeson |
| Norway | Skeianes | SK4 | 24211 | 6727785 | Anne Sverdrup-Thygeson |
| Norway | Skeianes | SK5 | 24286 | 6727809 | Anne Sverdrup-Thygeson |
| Norway | Steinknapp Øst | SO1 | 157912 | 6564176 | Anne Sverdrup-Thygeson |
| Norway | Steinknapp Øst | SO2 | 157780 | 6564330 | Anne Sverdrup-Thygeson |
| Norway | Steinknapp Øst | SO4 | 157747 | 6564385 | Anne Sverdrup-Thygeson |
| Norway | Steinknapp Øst | SO5 | 157739 | 6564401 | Anne Sverdrup-Thygeson |
| Norway | Steinknapp Øst | SO6 | 157770 | 6564326 | Anne Sverdrup-Thygeson |
| Norway | Steinknapp Vest | SV1 | 157012 | 6563991 | Anne Sverdrup-Thygeson |
| Norway | Steinknapp Vest | SV2 | 157003 | 6564000 | Anne Sverdrup-Thygeson |
| Norway | Steinknapp Vest | SV3 | 157005 | 6564009 | Anne Sverdrup-Thygeson |
| Norway | Steinknapp Vest | SV4 | 156965 | 6564090 | Anne Sverdrup-Thygeson |
| Norway | Tanum | TA1 | 247154 | 6648790 | Anne Sverdrup-Thygeson |
| Norway | Tomb | TO1 | 261734 | 6583346 | Anne Sverdrup-Thygeson |
| Norway | Vassbotn | VA1 | 208708 | 6558882 | Anne Sverdrup-Thygeson |
| Norway | Vemannsås | VE1 | 211336 | 6565841 | Anne Sverdrup-Thygeson |
| Norway | Vemannsås | VE2 | 211233 | 6565815 | Anne Sverdrup-Thygeson |
| Norway | Vemannsås | VE3 | 211277 | 6566151 | Anne Sverdrup-Thygeson |
| Norway | Vemannsås | VE4 | 211098 | 6565824 | Anne Sverdrup-Thygeson |
| Norway | Vemannsås | VE5 | 211174 | 6565846 | Anne Sverdrup-Thygeson |
| Norway | Veholt | VH1 | 188322 | 6568387 | Anne Sverdrup-Thygeson |
| Norway | Bjørnehula i Kjosdalen | BjVA1 | 84431 | 6463516 | Hanne Eik Pilskog |
| Norway | Brekkeseter | BrVE3 | 210489 | 6557857 | Hanne Eik Pilskog |
| Norway | Engene, Kjose | EnVE10 | 205985 | 6563697 | Hanne Eik Pilskog |
| Norway | Fuglevik | FuVE1 | 213582 | 6549028 | Hanne Eik Pilskog |
| Norway | Gangdalen mot Otra | GaVA1 | 85713 | 6471429 | Hanne Eik Pilskog |
| Norway | Gillsveien v/Gillsvann | GiVA1 | 90446 | 6470269 | Hanne Eik Pilskog |
| Norway | Håstøl | HaaAA5 | 106295 | 6502760 | Hanne Eik Pilskog |
| Norway | Håverstad | HaavAA9 | 99125 | 6505579 | Hanne Eik Pilskog |
| Norway | Håvaldsrød | HaaVE2 | 212459 | 6555682 | Hanne Eik Pilskog |
| Norway | Hushovd | HuAA3 | 107380 | 6524889 | Hanne Eik Pilskog |
| Norway | Hovland Ø | Hul3 | 98818 | 6514990 | Hanne Eik Pilskog |
| Norway | Kverndalen | KdAA13 | 110814 | 6476980 | Hanne Eik Pilskog |
| Norway | Kiste ved Lakssjø | KiTE1 | 204060 | 6578440 | Hanne Eik Pilskog |
| Norway | Lysebo | LyVE3 | 211936 | 6567273 | Hanne Eik Pilskog |
| Norway | Ødegården | OdVE2 | 206920 | 6548066 | Hanne Eik Pilskog |
| Norway | Øgården | OgVE8 | 209015 | 6559909 | Hanne Eik Pilskog |
| Norway | Retterholt | ReAA3 | 104232 | 6504430 | Hanne Eik Pilskog |
| Norway | Røsaker S | ROSK2 | 195184 | 6577850 | Hanne Eik Pilskog |
| Norway | Sekkebekk | SeAA5 | 110133 | 6475185 | Hanne Eik Pilskog |
| Norway | Skåre | SkAA3 | 101228 | 6515241 | Hanne Eik Pilskog |
| Norway | Skjærsjø | SkVE11 | 210731 | 6573043 | Hanne Eik Pilskog |
| Norway | Søndre Odbergsetra | SOVE3 | 209688 | 6578532 | Hanne Eik Pilskog |
| Norway | Stueåsen | StVE1 | 208784 | 6563384 | Hanne Eik Pilskog |
| Norway | Eg Sykehus | SyVA1 | 87590 | 6468677 | Hanne Eik Pilskog |
| Norway | Tjomsevann | TjVA1 | 79244 | 6462450 | Hanne Eik Pilskog |
| Norway | Tveitetjønnane SØ | TvAA1 | 103056 | 6481036 | Hanne Eik Pilskog |
| Norway | Tveitemyrane | TveAA2 | 105452 | 6481213 | Hanne Eik Pilskog |
| Norway | Vassbotnvannet | VaVE3 | 208308 | 6558526 | Hanne Eik Pilskog |
| Sweden | Sundsbro | SvLiSun1 | 545137 | 6466251 | Nicklas Jansson |
| Sweden | Sundsbro | SvLiSun2 | 545129 | 6466231 | Nicklas Jansson |
| Sweden | Sundsbro | SvLiSun3 | 545184 | 6466233 | Nicklas Jansson |
| Sweden | Sundsbro | SvLiSun5 | 545114 | 6466338 | Nicklas Jansson |
| Sweden | Sturefors-S | SvLiStS1 | 544876 | 6466295 | Nicklas Jansson |
| Sweden | Sturefors-S | SvLiStS3 | 544692 | 6466346 | Nicklas Jansson |
| Sweden | Sturefors-N | SvLiStN1 | 544764 | 6466347 | Nicklas Jansson |
| Sweden | Sturefors-N | SvLiStN2 | 544842 | 6466528 | Nicklas Jansson |
| Sweden | Sturefors-N | SvLiStN4 | 544801 | 6466539 | Nicklas Jansson |
| Sweden | Sturefors-N | SvLiStN5 | 544832 | 6466508 | Nicklas Jansson |
| Sweden | Hjorthägnet-N | SvLiHjN1 | 543306 | 6459665 | Nicklas Jansson |
| Sweden | Hjorthägnet-N | SvLiHjN2 | 543333 | 6459708 | Nicklas Jansson |
| Sweden | Hjorthägnet-N | SvLiHjN3 | 543375 | 6459679 | Nicklas Jansson |
| Sweden | Hjorthägnet-N | SvLiHjN4 | 543395 | 6459668 | Nicklas Jansson |
| Sweden | Hjorthägnet-N | SvLiHjN5 | 543415 | 6459679 | Nicklas Jansson |
| Sweden | Hjorthägnet-S | SvLiHjS1 | 543300 | 6459559 | Nicklas Jansson |
| Sweden | Hjorthägnet-S | SvLiHjS2 | 543336 | 6459548 | Nicklas Jansson |
| Sweden | Hjorthägnet-S | SvLiHjS3 | 543366 | 6459528 | Nicklas Jansson |
| Sweden | Hjorthägnet-S | SvLiHjS4 | 543364 | 6459532 | Nicklas Jansson |
| Sweden | Hjorthägnet-S | SvLiHjS5 | 543293 | 6459581 | Nicklas Jansson |
| Sweden | Bjärka äng | SvLiBjä1 | 544379 | 6459714 | Nicklas Jansson |
| Sweden | Bjärka äng | SvLiBjä2 | 544417 | 6459740 | Nicklas Jansson |
| Sweden | Bjärka äng | SvLiBjä3 | 544431 | 6459635 | Nicklas Jansson |
| Sweden | Bjärka äng | SvLiBjä4 | 544322 | 6459598 | Nicklas Jansson |
| Sweden | Bjärka äng | SvLiBjä5 | 544253 | 6459662 | Nicklas Jansson |
| Sweden | Fornhagen | SvLiFor1 | 543865 | 6459257 | Nicklas Jansson |
| Sweden | Fornhagen | SvLiFor2 | 543938 | 6459288 | Nicklas Jansson |
| Sweden | Fornhagen | SvLiFor3 | 543871 | 6459285 | Nicklas Jansson |
| Sweden | Fornhagen | SvLiFor4 | 543875 | 6459293 | Nicklas Jansson |
| Sweden | Fornhagen | SvLiFor5 | 543885 | 6459374 | Nicklas Jansson |
| Sweden | Orräng 1 | SvLiOrA1 | 550310 | 6461762 | Nicklas Jansson |
| Sweden | Orräng 1 | SvLiOrA2 | 550316 | 6461797 | Nicklas Jansson |
| Sweden | Orräng 1 | SvLiOrA3 | 550448 | 6461810 | Nicklas Jansson |
| Sweden | Orräng 1 | SvLiOrA4 | 550377 | 6461712 | Nicklas Jansson |
| Sweden | Orräng 1 | SvLiOrA5 | 550416 | 6461742 | Nicklas Jansson |
| Sweden | Orräng 2 | SvLiOrB1 | 550166 | 6461569 | Nicklas Jansson |
| Sweden | Orräng 2 | SvLiOrB2 | 550147 | 6461531 | Nicklas Jansson |
| Sweden | Orräng 2 | SvLiOrB3 | 550162 | 6461524 | Nicklas Jansson |
| Sweden | Orräng 2 | SvLiOrB4 | 550152 | 6461502 | Nicklas Jansson |
| Sweden | Orräng 2 | SvLiOrB5 | 550156 | 6461513 | Nicklas Jansson |
| Sweden | Orräng 3 | SvLiOrC2 | 550026 | 6461563 | Nicklas Jansson |
| Sweden | Orräng 3 | SvLiOrC3 | 549940 | 6461595 | Nicklas Jansson |
| Sweden | Orräng 3 | SvLiOrC4 | 549950 | 6461636 | Nicklas Jansson |
| Sweden | Orräng 3 | SvLiOrC5 | 549954 | 6461627 | Nicklas Jansson |
| Sweden | Skaggebo | SvLiSka1 | 543201 | 6457103 | Nicklas Jansson |
| Sweden | Skaggebo | SvLiSka2 | 543198 | 6457131 | Nicklas Jansson |
| Sweden | Skaggebo | SvLiSka3 | 543225 | 6457077 | Nicklas Jansson |
| Sweden | Skaggebo | SvLiSka4 | 543241 | 6457062 | Nicklas Jansson |
| Sweden | Skaggebo | SvLiSka5 | 543223 | 6457021 | Nicklas Jansson |
| Sweden | Labbenäs | SvLiLab1 | 539407 | 6463751 | Nicklas Jansson |
| Sweden | Labbenäs | SvLiLab2 | 539415 | 6463759 | Nicklas Jansson |
| Sweden | Labbenäs | SvLiLab3 | 539480 | 6463691 | Nicklas Jansson |
| Sweden | Labbenäs | SvLiLabextra | 539457 | 6463730 | Nicklas Jansson |
| Sweden | Labbenäs | SvLiLab4 | 539557 | 6463684 | Nicklas Jansson |
| Sweden | Labbenäs | SvLiLab5 | 539573 | 6463681 | Nicklas Jansson |
| Sweden | Labbenäs | SvLiLab6 | 539577 | 6463670 | Nicklas Jansson |
| Sweden | Brokind skolh. | SvLiBro1 | 539027 | 6452097 | Nicklas Jansson |
| Sweden | Brokind skolh. | SvLiBro2 | 539053 | 6451994 | Nicklas Jansson |
| Sweden | Brokind skolh. | SvLiBro3 | 539036 | 6451932 | Nicklas Jansson |
| Sweden | Brokind skolh. | SvLiBro4 | 539115 | 6451822 | Nicklas Jansson |
| Sweden | Brokind skolh. | SvLiBro5 | 538998 | 6451766 | Nicklas Jansson |
| Sweden | Sätra Humpen | SvLiSät1 | 544161 | 6461319 | Nicklas Jansson |
| Sweden | Sätra Humpen | SvLiSät2 | 544173 | 6461332 | Nicklas Jansson |
| Sweden | Sätra Humpen | SvLiSät3 | 544124 | 6461331 | Nicklas Jansson |
| Sweden | Sätra Humpen | SvLiSät4 | 544098 | 6461376 | Nicklas Jansson |
| Sweden | Sätra Humpen | SvLiSät5 | 543981 | 6460500 | Nicklas Jansson |
| Sweden | Långvassudde Ö | SvLiLåÖ1 | 546269 | 6466248 | Nicklas Jansson |
| Sweden | Långvassudde Ö | SvLiLåÖ2 | 546273 | 6466253 | Nicklas Jansson |
| Sweden | Långvassudde Ö | SvLiLåÖ3 | 546267 | 6466287 | Nicklas Jansson |
| Sweden | Långvassudde Ö | SvLiLåÖ4 | 546226 | 6466089 | Nicklas Jansson |
| Sweden | Långvassudde Ö | SvLiLåÖ5 | 546246 | 6466058 | Nicklas Jansson |
| Sweden | Långvassudde V | SvLiLåV1 | 546083 | 6466427 | Nicklas Jansson |
| Sweden | Långvassudde V | SvLiLåV3 | 546073 | 6466481 | Nicklas Jansson |
| Sweden | Långvassudde V | SvLiLåV5 | 546034 | 6466392 | Nicklas Jansson |
| Sweden | Biskopstorp | SvHaBis1 | 369494 | 6296135 | Nicklas Jansson |
| Sweden | Biskopstorp | SvHaBis2 | 369494 | 6296135 | Nicklas Jansson |
| Sweden | Biskopstorp | SvHaBis3 | 369494 | 6296135 | Nicklas Jansson |
| Sweden | Biskopstorp | SvHaBis4 | 369494 | 6296135 | Nicklas Jansson |
| Sweden | Biskopstorp | SvHaBis5 | 369494 | 6296135 | Nicklas Jansson |
| Sweden | Gässlösa | SvHaGäs4 | 348275 | 6336763 | Nicklas Jansson |
| Sweden | Gässlösa | SvHaGäs5 | 348275 | 6336763 | Nicklas Jansson |
| Sweden | Gässlösa | SvHaGäs6 | 348275 | 6336763 | Nicklas Jansson |
| Sweden | Hördalen | SvHaHör1 | 318724 | 6376467 | Nicklas Jansson |
| Sweden | Hördalen | SvHaHör2 | 318724 | 6376467 | Nicklas Jansson |
| Sweden | Hördalen | SvHaHör3 | 318724 | 6376467 | Nicklas Jansson |
| Sweden | Hördalen | SvHaHör6 | 318724 | 6376467 | Nicklas Jansson |
| Sweden | Stövlaberget | SvHaStö1 | 370454 | 6301154 | Nicklas Jansson |
| Sweden | Stövlaberget | SvHaStö2 | 370454 | 6301154 | Nicklas Jansson |
| Sweden | Stövlaberget | SvHaStö3 | 370454 | 6301154 | Nicklas Jansson |
| Sweden | Stövlaberget | SvHaStö5 | 370454 | 6301154 | Nicklas Jansson |
| Sweden | Särö | SvHaSär1 | 315806 | 6377854 | Nicklas Jansson |
| Sweden | Särö | SvHaSär2 | 315806 | 6377854 | Nicklas Jansson |
| Sweden | Särö | SvHaSär3 | 315806 | 6377854 | Nicklas Jansson |
| Sweden | Särö | SvHaSär5 | 315806 | 6377854 | Nicklas Jansson |
| Sweden | Särö | SvHaSär6 | 315806 | 6377854 | Nicklas Jansson |
| Sweden | Tjolöholm | SvHaTjo1 | 324942 | 6365970 | Nicklas Jansson |
| Sweden | Tjolöholm | SvHaTjo2 | 324942 | 6365970 | Nicklas Jansson |
| Sweden | Tjolöholm | SvHaTjo3 | 324942 | 6365970 | Nicklas Jansson |
| Sweden | Tjolöholm | SvHaTjo4 | 324942 | 6365970 | Nicklas Jansson |
| Sweden | Åkraberga | SvHaÅkr1 | 332990 | 6349744 | Nicklas Jansson |
| Sweden | Åkraberga | SvHaÅkr2 | 332990 | 6349744 | Nicklas Jansson |
| Sweden | Åkraberga | SvHaÅkr5 | 332990 | 6349744 | Nicklas Jansson |
| Sweden | Åkraberga | SvHaÅkr6 | 332990 | 6349744 | Nicklas Jansson |
| Sweden | Åkersberga | Akersberga1 | 686091 | 6599064 | Mats Jonsell |
| Sweden | Åkersberga | Akersberga2 | 686031 | 6599097 | Mats Jonsell |
| Sweden | Åkersberga | Akersberga3 | 686001 | 6599409 | Mats Jonsell |
| Sweden | Åkersberga | Akersberga4 | 685946 | 6599366 | Mats Jonsell |
| Sweden | Biskops-Arnö | Arno1 | 640808 | 6616772 | Mats Jonsell |
| Sweden | Biskops-Arnö | Arno2 | 640758 | 6616826 | Mats Jonsell |
| Sweden | Biskops-Arnö | Arno3 | 640733 | 6616854 | Mats Jonsell |
| Sweden | Biskops-Arnö | Arno4 | 640989 | 6616542 | Mats Jonsell |
| Sweden | Biskops-Arnö | Biskops_Arno1 | 640808 | 6616772 | Mats Jonsell |
| Sweden | Biskops-Arnö | Biskops_Arno2 | 640758 | 6616826 | Mats Jonsell |
| Sweden | Biskops-Arnö | Biskops_Arno3 | 640989 | 6616542 | Mats Jonsell |
| Sweden | Biskops-Arnö | Biskops_Arno4 | 641011 | 6616511 | Mats Jonsell |
| Sweden | Drottningholm | Drottningholm5 | 663583 | 6578978 | Mats Jonsell |
| Sweden | Drottningholm | Drottningholm6 | 663472 | 6579182 | Mats Jonsell |
| Sweden | Drottningholm | Drottningholm7 | 663410 | 6579189 | Mats Jonsell |
| Sweden | Drottningholm | Drottningholm8 | 663813 | 6579120 | Mats Jonsell |
| Sweden | Edeby ekhagar | Edeby_ekhagar1 | 662777 | 6577252 | Mats Jonsell |
| Sweden | Edeby ekhagar | Edeby_ekhagar2 | 662848 | 6577280 | Mats Jonsell |
| Sweden | Edeby ekhagar | Edeby_ekhagar3 | 662926 | 6577247 | Mats Jonsell |
| Sweden | Edeby ekhagar | Edeby_ekhagar4 | 662842 | 6577130 | Mats Jonsell |
| Sweden | Ekudden | Ekudden1 | 679620 | 6572827 | Mats Jonsell |
| Sweden | Ekudden | Ekudden2 | 679588 | 6572647 | Mats Jonsell |
| Sweden | Ekudden | Ekudden3 | 679638 | 6572701 | Mats Jonsell |
| Sweden | Ekudden | Ekudden4 | 679618 | 6572717 | Mats Jonsell |
| Sweden | Fånö | F1 | 634890 | 6605446 | Mats Jonsell |
| Sweden | Fånö | F2 | 634759 | 6605506 | Mats Jonsell |
| Sweden | Fånö | F3 | 634805 | 6605639 | Mats Jonsell |
| Sweden | Fånö | F4 | 634494 | 6606038 | Mats Jonsell |
| Sweden | Hågadalen | Hagadalen1 | 645956 | 6633895 | Mats Jonsell |
| Sweden | Hågadalen | Hagadalen2 | 645617 | 6634002 | Mats Jonsell |
| Sweden | Hågadalen | Hagadalen3 | 645820 | 6633534 | Mats Jonsell |
| Sweden | Hågadalen | Hagadalen4 | 645260 | 6635470 | Mats Jonsell |
| Sweden | Harparbollund | Harparbol1 | 667947 | 6641276 | Mats Jonsell |
| Sweden | Harparbollund | Harparbol2 | 668018 | 6641231 | Mats Jonsell |
| Sweden | Harparbollund | Harparbol3 | 668065 | 6641285 | Mats Jonsell |
| Sweden | Harparbollund | Harparbol4 | 668085 | 6641314 | Mats Jonsell |
| Sweden | Hjulsta | Hjul1 | 613446 | 6601220 | Mats Jonsell |
| Sweden | Hjulsta | Hjul2 | 613864 | 6601473 | Mats Jonsell |
| Sweden | Hjulsta | Hjul3 | 613862 | 6601194 | Mats Jonsell |
| Sweden | Hjulsta | Hjul4 | 613917 | 6601294 | Mats Jonsell |
| Sweden | Kalhäll. Lädersättra | KL1 | 659580 | 6593086 | Mats Jonsell |
| Sweden | Kalhäll. Lädersättra | KL2 | 659602 | 6593137 | Mats Jonsell |
| Sweden | Kalhäll. Lädersättra | KL3 | 659709 | 6593097 | Mats Jonsell |
| Sweden | Kalhäll. Lädersättra | KL4 | 659755 | 6593054 | Mats Jonsell |
| Sweden | Kristineholm | Kristineholm1 | 694231 | 6639502 | Mats Jonsell |
| Sweden | Kristineholm | Kristineholm2 | 694266 | 6639535 | Mats Jonsell |
| Sweden | Kristineholm | Kristineholm3 | 694617 | 6639777 | Mats Jonsell |
| Sweden | Kristineholm | Kristineholm4 | 694533 | 6639626 | Mats Jonsell |
| Sweden | Krusenberg | Krus1 | 648520 | 6624922 | Mats Jonsell |
| Sweden | Krusenberg | Krus2 | 648710 | 6625075 | Mats Jonsell |
| Sweden | Krusenberg | Krus3 | 648564 | 6625040 | Mats Jonsell |
| Sweden | Krusenberg | Krus4 | 648498 | 6625281 | Mats Jonsell |
| Sweden | Krusenberg | Krus5 | 648600 | 6625607 | Mats Jonsell |
| Sweden | Näsudden | Nasudden1 | 660635 | 6641834 | Mats Jonsell |
| Sweden | Näsudden | Nasudden2 | 660588 | 6641757 | Mats Jonsell |
| Sweden | Näsudden | Nasudden3 | 660633 | 6641591 | Mats Jonsell |
| Sweden | Näsudden | Nasudden4 | 660538 | 6641610 | Mats Jonsell |
| Sweden | Norrmalma | Norrmalma1 | 704715 | 6638551 | Mats Jonsell |
| Sweden | Norrmalma | Norrmalma2 | 704743 | 6638640 | Mats Jonsell |
| Sweden | Norrmalma | Norrmalma3 | 704681 | 6638520 | Mats Jonsell |
| Sweden | Norrmalma | Norrmalma4 | 704615 | 6638680 | Mats Jonsell |
| Sweden | Olivedal | Olivedal1 | 662444 | 6637377 | Mats Jonsell |
| Sweden | Olivedal | Olivedal2 | 662382 | 6637377 | Mats Jonsell |
| Sweden | Olivedal | Olivedal3 | 662293 | 6637402 | Mats Jonsell |
| Sweden | Olivedal | Olivedal4 | 662328 | 6637459 | Mats Jonsell |
| Sweden | Parnassen | Parnassen1 | 634496 | 6615423 | Mats Jonsell |
| Sweden | Parnassen | Parnassen3 | 634091 | 6615256 | Mats Jonsell |
| Sweden | Parnassen | Parnassen4 | 633974 | 6615305 | Mats Jonsell |
| Sweden | Salsta | Salsta1 | 652388 | 6659194 | Mats Jonsell |
| Sweden | Salsta | Salsta2 | 652442 | 6658968 | Mats Jonsell |
| Sweden | Salsta | Salsta3 | 653131 | 6657896 | Mats Jonsell |
| Sweden | Salsta | Salsta4 | 653031 | 6657949 | Mats Jonsell |
| Sweden | Sickla udde | Sickla_udde1 | 677076 | 6578012 | Mats Jonsell |
| Sweden | Sickla udde | Sickla_udde2 | 676987 | 6578118 | Mats Jonsell |
| Sweden | Sickla udde | Sickla_udde3 | 676985 | 6578171 | Mats Jonsell |
| Sweden | Sickla udde | Sickla_udde4 | 677164 | 6578010 | Mats Jonsell |
| Sweden | Tyska botten | Tyska_botten1 | 664260 | 6581324 | Mats Jonsell |
| Sweden | Tyska botten | Tyska_botten2 | 664202 | 6581377 | Mats Jonsell |
| Sweden | Tyska botten | Tyska_botten3 | 664099 | 6581406 | Mats Jonsell |
| Sweden | Tyska botten | Tyska_botten4 | 664053 | 6581401 | Mats Jonsell |
| Sweden | Vik | Vik1 | 639222 | 6624858 | Mats Jonsell |
| Sweden | Vik | Vik2 | 639257 | 6625060 | Mats Jonsell |
| Sweden | Vik | Vik3 | 639077 | 6625219 | Mats Jonsell |
| Sweden | Vik | Vik4 | 638915 | 6625049 | Mats Jonsell |
