# Supplementary material for: Specialists in ancient trees are more affected by climate than generalists
Source: Ecol Evol. 2015 Nov 17;5(23):5632–41. doi: 10.1002/ece3.1799 (PMC4813105; doi:10.1002/ece3.1799)
Supplement: Supplementary file 2 — Table S2. Categorization of dead wood inhabiting beetles species according to oak association and primary geographical distribution. [file ECE3-5-5632-s002.docx]

Table S2. Categorization of dead wood inhabiting beetles species according to oak association and primary geographical distribution. Oak association follows The Saproxylic Database (http://radon.uio.no/WDD/Login.aspx?ReturnUrl=%2fwdd%2fDefault.aspx) with specialists defined as species marked with p for oak or with only one tree association (x for oak). Generalists include all other species associated with oak (x for oak). The authors Mats Jonsell (MJ) and Niclas Jansson (NJ) also noted divergence between The Saproxylic database and their field experience. The species are according to Löbl, I. & A. Smetana 2003-2012. Catalogue of Palaearctic Coleoptera Vol I-VIII. – Apollo Books, Stenstrup, Denmark.

| **Family** | **Name** | **Oak association used** | **Oak association divergence MJ and NJ** | **Primary**  **geographical**  **distribution** |
| --- | --- | --- | --- | --- |
| Histeridae |  |  |  |  |
|  | *Plegaderus caesus* (Herbst, 1792) | Generalist | - | Southern |
|  | *Gnathoncus nannetensis* (Marseul, 1862) | Generalist | - | Northern |
|  | *Dendrophilus punctatus* (Herbst, 1792) | Generalist | - | Northern |
|  | *Paromalus flavicornis* (Herbst, 1792) | Generalist | - | Southern |
| Leiodidae |  |  |  |  |
|  | *Nemadus colonoides* (Kraatz, 1851) | Specialist | Generalist | Northern |
| Staphylinidae | |  |  |  |
|  | *Stenichnus godarti* (Latreille, 1806) | Specialist | Generalist | Southern |
|  | *Stenichnus bicolor* (Denny, 1825) | Generalist | - | Northern |
|  | *Scydmaenus hellwigii* (Herbst, 1792) | Specialist | Generalist | Southern |
|  | *Velleius dilatatus* (Fabricius, 1787) | Generalist | - | Southern |
|  | *Quedius microps* (Gravenhorst, 1847) | Generalist | - | Southern |
|  | *Quedius mesomelinus* (Marsham, 1802) | Generalist | - | Other |
|  | *Quedius maurus* (Sahlberg, 1830) | Generalist | - | Northern |
|  | *Quedius cruentus* (Olivier, 1795) | Generalist | - | Southern |
|  | *Quedius invreae* Gridelli, 1924 | Specialist | Generalist | Southern |
|  | *Quedius brevicornis* Thomson, 1860 | Generalist | - | Southern |
|  | *Quedius scitus* (Gravenhorst, 1806) | Specialist | Generalist | Southern |
|  | *Quedius xanthopus* Erichson, 1839 | Generalist | - | Northern |
|  | *Quedius plagiatus* (Mannerheim, 1843) | Generalist | - | Northern |
|  | *Euplectus brunneus* (Grimmer, 1841) | Generalist | - | Southern |
|  | *Euplectus nanus* (Reichenbach, 1816) | Generalist | - | Northern |
|  | *Euplectus piceus* Motschulsky, 1835 | Generalist | - | Southern |
|  | *Euplectus punctatus* Mulsant, 1861 | Generalist | - | Northern |
|  | *Euplectus karstenii* (Reichenbach, 1816) | Generalist | - | Northern |
|  | *Euplectus mutator* Fauvel, 1895 | Generalist | - | Northern |
|  | *Batrisodes adnexus* (Hampe, 1863) | Specialist | Generalist | Southern |
|  | *Batrisodes delaporti* (Aubé, 1833) | Specialist | Generalist | Southern |
|  | *Batrisodes venustus* (Reichenbach, 1816) | Generalist | - | Southern |
|  | *Bibloporus bicolor* (Denny, 1825) | Generalist | - | Northern |
|  | *Bibloporus minutus* Raffray, 1914 | Generalist | - | Northern |
|  | *Hapalaraea pygmaea* (Paykull, 1800) | Generalist | Not in Oak | Northern |
| Trogidae |  |  |  |  |
|  | *Trox scaber* (Linnaeus, 1767) | Generalist | - | Southern |
| Scarabaeidae | |  |  |  |
|  | O*smoderma eremita* (Scopoli, 1763) | Specialist | Generalist | Southern |
|  | *Gnorimus nobilis* (Linnaeus, 1758) | Generalist | - | Southern |
|  | *Protaetia marmorata* (Fabricius, 1792) | Specialist | Generalist | Southern |
|  | *Protaetia metallica* (Herbst, 1786) | Generalist | - | Southern |
| Lucanidae |  |  |  |  |
|  | *Sinodendron cylindricum* (Linnaeus, 1758) | Generalist | - | Southern |
|  | *Platycerus caraboides* (Linnaeus, 1758) | Generalist | - | Southern |
| Scirtidae |  |  |  |  |
|  | *Prionocyphon serricornis* (Müller, 1821) | Generalist | - | Southern |
| Buprestidae |  |  |  |  |
|  | *Agrilus angustulus* (Illiger, 1803) | Generalist | Specialist | Southern |
|  | *Agrilus sulcicollis* Lacordaire, 1835 | Specialist | - | Southern |
| Elateridae |  |  |  |  |
|  | *Stenagostus rhombeus* (Olivier, 1790) | Generalist | - | Southern |
|  | *Denticollis linearis* (Linnaeus, 1758) | Generalist | - | Northern |
|  | *Crepidophorus mutilatus* (Rosenhauer, 1847) | Generalist | - | Northern |
|  | *Hypoganus inunctus* (Lacordaire, 1835) | Generalist | - | Southern |
|  | *Calambus bipustulatus* (Linnaeus, 1767) | Generalist | - | Southern |
|  | *Procraerus tibialis* (Lacordaire, 1835) | Generalist | Specialist | Southern |
|  | *Ampedus cardinalis* (Schiödte, 1865) | Specialist | - | Other |
|  | *Ampedus hjorti* (Rye, 1905) | Specialist | - | Other |
|  | *Ampedus nigroflavus* (Goeze, 1777) | Generalist | - | Southern |
|  | *Ampedus praeustus* (Fabricius, 1792) | Generalist | - | Other |
|  | *Ampedus pomorum* (Herbst, 1784) | Generalist | - | Northern |
|  | *Ampedus balteatus* (Linnaeus, 1758) | Generalist | - | Northern |
|  | *Ampedus nigrinus* (Herbst, 1784) | Specialist | - | Northern |
|  | *Elater ferrugineus* Linnaeus, 1758 | Specialist | Generalist | Other |
|  | *Melanotus villosus* (Geoffroy, 1785) | Generalist | - | Southern |
| Eucnemidae |  |  |  |  |
|  | *Melasis buprestoides* (Linnaeus, 1760) | Generalist | - | Southern |
|  | *Xylophilus corticalis* (Paykull, 1800) | Generalist | - | Other |
| Dermestidae |  |  |  |  |
|  | *Attagenus pellio* (Linnaeus, 1758) | Generalist | - | Other |
|  | *Megatoma undata* (Linnaeus, 1758) | Generalist | - | Northern |
|  | *Ctesias serra* (Fabricius, 1792) | Generalist | - | Northern |
|  | *Anthrenus scrophulariae* (Linnaeus, 1758) | Generalist | - | Southern |
|  | *Anthrenus museorum* (Linnaeus, 1760) | Generalist | - | Southern |
| Ptinidae |  |  |  |  |
|  | *Ptinomorphus imperialis* (Linnaeus, 1767) | Generalist | - | Southern |
|  | *Ptinus rufipes* Olivier, 1790 | Specialist | - | Southern |
|  | *Ptinus fur* (Linnaeus, 1758) | Generalist | - | Other |
|  | *Ptinus subpillosus* Sturm, 1837 | Specialist | Generalist | Southern |
|  | *Grynobius planus* (Fabricius, 1787) | Generalist | - | Northern |
|  | *Xestobium rufovillosum* (Degeer, 1774) | Specialist | - | Other |
|  | *Oligomerus brunneus* (Olivier, 1790) | Specialist | - | Southern |
|  | *Hadrobregmus pertinax* (Linnaeus, 1758) | Generalist | - | Northern |
|  | *Gastrallus immarginatus* (Müller, 1821) | Generalist | Specialist | Southern |
|  | *Cacotemnus rufipes* (Fabricius, 1792) | Generalist | - | Northern |
|  | *Hemicoelus canaliculatus* (Thomson, 1863) | Generalist | - | Northern |
|  | *Ptilinus pectinicornis* (Linnaeus, 1758) | Generalist | - | Southern |
|  | *Xyletinus pectinatus* (Fabricius, 1792) | Specialist | Generalist | Northern |
|  | *Dorcatoma flavicornis* (Fabricius, 1792) | Generalist | Specialist | Northern |
|  | *Dorcatoma chrysomelina* Sturm, 1837 | Specialist | - | Northern |
|  | *Dorcatoma dresdensis* Herbst, 1792 | Generalist | - | Northern |
| Lymexylidae |  |  |  |  |
|  | *Elateroides dermestoides* (Linnaeus, 1760) | Generalist | - | Northern |
|  | *Lymexylon navale* (Linnaeus, 1758) | Specialist | Generalist | Southern |
| Malachiidae |  |  |  |  |
|  | *Hypebaeus flavipes* (Fabricius, 1787) | Generalist | - | Southern |
|  | *Malachius bipustulatus* (Linnaeus, 1758) | Generalist | - | Other |
| Trogossitidae | |  |  |  |
|  | *Grynocharis oblonga* (Linnaeus, 1758) | Generalist | - | Northern |
| Cleridae |  |  |  |  |
|  | *Tillus elongatus* (Linnaeus, 1758) | Generalist | - | Southern |
| Dasytidae |  |  |  |  |
|  | *Trichoceble memnonia* (Kiesenwetter, 1861) | Generalist | - | Southern |
| Cryptophagidae | |  |  |  |
|  | *Cryptophagus confusus* Bruce, 1934 | Generalist | - | Northern |
|  | *Cryptophagus labilis* Erichson, 1846 | Generalist | - | Northern |
|  | *Cryptophagus populi* Paykull, 1800 | Generalist | - | Northern |
|  | *Cryptophagus quercinus* Kraatz, 1852 | Generalist | - | Northern |
|  | *Cryptophagus badius* Sturm, 1845 | Generalist | - | Northern |
|  | *Cryptophagus dentatus* (Herbst, 1793) | Generalist | - | Southern |
|  | *Cryptophagus denticulatus* Heer, 1841 | Generalist | - | Northern |
|  | *Cryptophagus scanicus* (Linnaeus, 1758) | Generalist | - | Northern |
|  | *Cryptophagus scutellatus* Newman, 1834 | Generalist | - | Other |
|  | *Cryptophagus setulosus* Sturm, 1845 | Generalist | - | Northern |
|  | *Atomaria morio* Kolenati, 1846 | Generalist | - | Southern |
|  | *Atomaria umbrina* (Gyllenhal, 1827) | Generalist | - | Northern |
| Erotylidae |  |  |  |  |
|  | *Dacne bipustulata* (Thunberg, 1781) | Generalist | - | Northern |
|  | *Triplax russica* (Linnaeus, 1758) | Generalist | - | Northern |
|  |  |  |  |  |
|  | *Cerylon histeroides* (Fabricius, 1792) | Generalist | - | Northern |
|  | *Cerylon ferrugineum* Stephens, 1830 | Generalist | - | Northern |
| Endomychidae | |  |  |  |
|  | *Mycetaea subterranea* (Fabricius, 1801) | Generalist | - | Southern |
| Zopheridae |  |  |  |  |
|  | *Colydium filiforme* Fabricius, 1792 | Specialist | - | Southern |
|  | *Synchita humeralis* (Fabricius, 1792) | Generalist | - | Other |
| Mycetophagidae | |  |  |  |
|  | *Triphyllus bicolor* (Fabricius, 1777) | Generalist | - | Southern |
|  | *Litargus connexus* (Geoffroy, 1785) | Generalist | - | Other |
|  | *Mycetophagus piceus* (Fabricius, 1777) | Specialist | Generalist | Northern |
| Tenebrionidae | |  |  |  |
|  | *Mycetophagus multipunctatus* Fabricius, 1792 | Generalist | - | Northern |
| Salpingidae |  |  |  |  |
|  | *Salpingus planirostris* (Fabricius, 1787) | Generalist | - | Southern |
|  | *Salpingus ruficollis* (Linnaeus, 1760) | Generalist | - | Northern |
| Aderidae |  |  |  |  |
|  | *Aderus populneus* (Creutzer, 1796) | Generalist | - | Southern |
|  | *Euglenes oculatus* (Paykull, 1798) | Specialist | - | Northern |
|  | *Euglenes pygmaeus* (Degeer, 1774) | Specialist | - | Other |
| Tenebrionidae | |  |  |  |
|  | *Uloma culinaris* (Linnaeus, 1758) | Generalist | - | Southern |
|  | *Tenebrio opacus* Duftschmid, 1812 | Specialist | - | Southern |
|  | *Tenebrio molitor* Linnaeus, 1758 | Generalist | - | Southern |
|  | *Eledona agricola* (Herbst, 1783) | Specialist | - | Other |
|  | *Palorus depressus* (Fabricius, 1790) | Specialist | Generalist | Southern |
|  | *Allecula morio* (Fabricius, 1787) | Generalist | - | Northern |
|  | *Prionychus ater* (Fabricius, 1775) | Generalist | - | Southern |
|  | *Pseudocistela ceramboides* (Linnaeus, 1758) | Generalist | - | Southern |
|  | *Mycetochara humeralis* (Fabricius, 1787) | Generalist | - | Southern |
|  | *Mycetochara flavipes* (Fabricius, 1792) | Generalist | - | Northern |
|  | *Mycetochara maura* (Fabricius, 1792) | Generalist | - | Southern |
|  | Corticeus fasciatus (Fabricius, 1790) | Specialist | - | Southern |
|  | *Diaperis boleti* (Linnaeus, 1758) | Generalist | - | Southern |
|  | *Pentaphyllus testaceus* (Hellwig, 1792) | Specialist | - | Southern |
| Scraptiidae |  |  |  |  |
|  | *Scraptia fuscula* Müller, 1821 | Specialist | - | Southern |
| Tetratomidae | |  |  |  |
|  | *Hallomenus binotatus* (Quensel, 1790) | Generalist | - | Other |
| Melandryidae | |  |  |  |
|  | *Orchesia fasciata* (Illiger, 1798) | Generalist | - | Northern |
|  | *Orchesia micans* (Panzer, 1793) | Generalist | - | Northern |
|  | *Orchesia minor* Walker, 1837 | Generalist | - | Northern |
|  | *Orchesia undulata* Kraatz, 1853 | Generalist | - | Southern |
|  | *Phloiotrya rufipes* (Gyllenhal, 1810) | Generalist | - | Southern |
|  | *Conopalpus testaceus* (Olivier, 1790) | Generalist | Specialist | Southern |
| Cerambycidae | |  |  |  |
|  | *Rhagium mordax* (Degeer, 1775) | Generalist | - | Northern |
|  | *Rhagium inquisitor* (Linnaeus, 1758) | Generalist | Not in Oak | Other |
|  | *Stenocorus meridianus* (Linnaeus, 1758) | Generalist | - | Southern |
|  | *Grammoptera ruficornis* (Fabricius, 1781) | Generalist | - | Southern |
|  | *Alosterna tabacicolor* (Degeer, 1775) | Specialist | Generalist | Other |
|  | *Anoplodera sexguttata* (Fabricius, 1775) | Specialist | - | Southern |
|  | *Leptura quadrifasciata* Linnaeus, 1758 | Generalist | - | Northern |
|  | *Stictoleptura maculicornis* (Degeer, 1775) | Generalist | - | Southern |
|  | *Rutpela maculata* (Poda, 1761) | Specialist | - | Southern |
|  | *Oxymirus cursor* (Linnaeus, 1758) | Generalist | - | Northern |
|  | *Phymatodes testaceus* (Linnaeus, 1758) | Generalist | - | Southern |
|  | *Clytus arietis* (Linnaeus, 1758) | Specialist | Generalist | Southern |
|  | *Plagionotus detritus* (Linnaeus, 1758) | Specialist | - | Southern |
|  | *Leiopus nebulosus* (Linnaeus, 1758) | Specialist | Generalist | Southern |
|  | *Stenostola dubia* (Laicharting, 1784) | Generalist | - | Southern |
| Curculionidae | |  |  |  |
|  | *Rhyncolus ater* (Linnaeus, 1758) | Generalist | - | Northern |
|  | *Rhyncolus sculpturatus* Waltl, 1839 | Generalist | - | Other |
