# Supplementary material for: Specialists in ancient trees are more affected by climate than generalists
Source: Ecol Evol. 2015 Nov 17;5(23):5632–41. doi: 10.1002/ece3.1799 (PMC4813105; doi:10.1002/ece3.1799)
Supplement: Supplementary file 3 — Table S3. Results of backwards stepwise selection of six models all including four predictor variables: Temperature, Precipitation, Oak Circumference and Openness around trees. [file ECE3-5-5632-s003.docx]

Table S3. Results of backwards stepwise selection of six models all including four predictor variables: Temperature, Precipitation, Oak Circumference and Openness around trees. The analysis is performed in R using function glmer from package lme4 with a Poisson error distribution, log-link function and Nelder-Mead optimizer from package nloptr. The backward selection is based on Akaike’s Information Criterion and the drop1 function.

| Variable | Estimate | Std. Error | Z value | *p*-value |
| --- | --- | --- | --- | --- |
|  |  |  |  |  |
| *Response: Northern Specialists*  *AIC Final Model: 769.5 (Initial: 774.1)*  Intercept  Circumference  Temperature | -0.186  0.213  0.425 | 0.201  0.061  0.114 | -0.92  3.48  3.74 | 0.356  <0.001  <0.001 |
| *Response: Southern Specialists*  *AIC Final Model: 801.4 (Initial: 804.8)*  Intercept  Circumference  Precipitation | -0.024  0.107  -0.316 | 0.073  0.062  0.092 | -0.32  1.74  -3.44 | 0.748  0.082  <0.001 |
| *Response: Ubiquitous Specialists*  *AIC Final Model: 634.8 (Initial: 637.7)*  Intercept  Circumference  Precipitation | -0.537  0.348  -0.618 | 0.085  0.065  0.144 | -6.34  5.38  -4.30 | <0.001  <0.001  <0.001 |
|  |  |  |  |  |
| *Response: Northern Generalists*  *AIC Final Model: 1347.7 (Initial: 1352.9)*  Intercept  Circumference | 1.272  0.101 | 0.068  0.038 | 18.61  2.64 | <0.001  0.008 |
| *Response: Southern Generalists*  *AIC Final Model: 1143.5 (Initial: 1146.5)*  Intercept  Circumference | 0.738  0.154 | 0.222  0.043 | 3.33  3.60 | <0.001  <0.001 |
| *Response: Ubiquitous Generalists*  *AIC Final Model: 584.1 (Initial: 590.2)*  Intercept  Precipitation | -0.592  -0.167 | 0.077  0.097 | -7.68  -1.72 | <0.001  0.086 |
